# Os Melhores Artigos do Ano 2021 nos *Arquivos Brasileiros de Cardiologia* e na *Revista Portuguesa de Cardiologia*

**DOI:** 10.36660/abc.20220312

**Published:** 2022-07-07

**Authors:** Ricardo Fontes-Carvalho, Gláucia Maria Moraes de Oliveira, Pedro Gonçalves-Teixeira, Carlos Eduardo Rochitte, Nuno Cardim

**Affiliations:** 1 Centro Hospitalar de Vila Nova de Gaia/Espinho Departamento de Cardiologia Vila Nova de Gaia Espinho Portugal Departamento de Cardiologia – Centro Hospitalar de Vila Nova de Gaia/Espinho, Vila Nova de Gaia/Espinho – Portugal; 2 University of Porto Faculty of Medicine Cardiovascular Research Center Porto Portugal Cardiovascular Research Center (UniC), Faculty of Medicine, University of Porto, Porto, Portugal; 3 Universidade Federal do Rio de Janeiro Faculdade de Medicina Rio de Janeiro RJ Brasil Faculdade de Medicina – Universidade Federal do Rio de Janeiro, Rio de Janeiro, RJ – Brasil; 4 Universidade Federal do Rio de Janeiro Instituto do Coração Edson Saad Rio de Janeiro RJ Brasil Instituto do Coração Edson Saad – Universidade Federal do Rio de Janeiro, Rio de Janeiro, RJ – Brasil; 5 Universidade de São Paulo Faculdade de Medicina Instituto do Coração São Paulo SP Brasil Instituto do Coração (InCor) – Faculdade de Medicina da Universidade de São Paulo, São Paulo, SP – Brasil; 6 Hospital do Coração São Paulo SP Brasil Hospital do Coração (HCOR), São Paulo, SP – Brasil; 7 Hospital da Luz Lisboa Portugal Hospital da Luz, Lisboa – Portugal

**Keywords:** Brasil, Cooperação Técnica/tendências, Disseminação da Informação, Doenças Cardiovasculares, Fator de Impacto

## Introdução

Anualmente, a *Revista Portuguesa de Cardiologia* (RPC) e os *Arquivos Brasileiros de Cardiologia* (ABC) têm publicado em conjunto um artigo especial com a seleção dos seus melhores trabalhos originais.^1–3^ Ao longo dos anos, foi possível observar a grande qualidade das publicações nas duas revistas, o que mostra o dinamismo da investigação cardiovascular nos países de língua portuguesa.

Dando seguimento a essa “tradição”, os corpos editoriais da RPC e dos ABC voltaram a se reunir para selecionar a lista dos seus dez melhores artigos publicados em 2021 (Tabelas 1 e 2). Mais uma vez, o ano de 2021 foi marcado pelo impacto da pandemia de COVID-19. Contudo, as duas revistas publicaram excelentes trabalhos em todas as áreas da Medicina Cardiovascular, desde a prevenção cardiovascular até à insuficiência cardíaca, incluindo uma primorosa seleção de artigos sobre a pandemia de COVID-19 nas populações brasileira e portuguesa. Cabe ressaltar que a tarefa de eleger os melhores trabalhos publicados ao longo de cada ano é complexa, devido à elevada qualidade das publicações em ambos os periódicos.

**Tabela 1:** Lista com a seleção dos dez melhores artigos publicados nos *Arquivos Brasileiros de Cardiologia* em 2021

Links	Autor e título do artigo
https://abccardiol.org/wp-content/uploads/articles_xml/0066-782X-abc-117-01-0091/0066-782X-abc-117-01-0091.x44344.pdf	Alves et al.
	Relação entre Resposta Imune Inata do Receptor Toll-Like-4 (TLR-4) e o Processo Fisiopatológico da Cardiomiopatia da Obesidade
	Relationship between Innate Immune Response Toll-Like Receptor 4 (TLR-4) and the Pathophysiological Process of Obesity Cardiomyopathy
https://abccardiol.org/wp-content/uploads/articles_xml/0066-782X-abc-116-06-1091/0066-782X-abc-116-06-1091.x44344.pdf	Morais et al.
	Performance Diagnóstica da FFR por Angiotomografia de Coronárias através de Software Baseado em Inteligência Artificial
	Diagnostic Performance of a Machine Learning-Based CT-Derived FFR in Detecting Flow-Limiting Stenosis
https://abccardiol.org/wp-content/uploads/articles_xml/0066-782X-abc-116-03-0466/0066-782X-abc-116-03-0466.x44344.pdf	Matos et al.
	O Escore Gensini e a Carga Trombótica Adicionam Valor Preditivo ao Escore SYNTAX na Detecção de No-Reflow após Infarto do Miocárdio
	Gensini Score and Thrombus Burden Add Predictive Value to the SYNTAX Score in Detecting No-Reflow after Myocardial Infarction
https://abccardiol.org/wp-content/uploads/articles_xml/0066-782X-abc-117-05-0944/0066-782X-abc-117-05-0944.x44344.pdf	Santos et al.
	Mortalidade por Insuficiência Cardíaca e Desenvolvimento Socioeconômico no Brasil, 1980 a 2018
	Mortality Due to Heart Failure and Socioeconomic Development in Brazil between 1980 and 2018
https://abccardiol.org/wp-content/uploads/articles_xml/0066-782X-abc-117-03-0426/0066-782X-abc-117-03-0426.x44344.pdf	Santos et al.
	Diagnóstico de Fibrilação Atrial na Comunidade Utilizando Eletrocardiograma e Autorrelato: Análise Transversal do ELSA-Brasil
	Atrial Fibrillation Diagnosis using ECG Records and Self-Report in the Community: Cross-Sectional Analysis from ELSA-Brasil
https://abccardiol.org/wp-content/uploads/articles_xml/0066-782X-abc-116-02-0219/0066-782X-abc-116-02-0219.x44344.pdf	Mendes et al.
	Resultados Clínicos e Hemodinâmicos de Longo Prazo após o Transplante de Coração em Pacientes Pré-Tratados com Sildenafil
	Long-Term Clinical and Hemodynamic Outcomes after Heart Transplantation in Patients Pre-Treated with Sildenafil.
https://abccardiol.org/wp-content/uploads/articles_xml/1678-4170-abc-116-04-0695/1678-4170-abc-116-04-0695.x44344.pdf	Oliveira et al.
	Acesso à Terapia de Reperfusão e Mortalidade em Mulheres com Infarto Agudo do Miocárdio com Supradesnivelamento do Segmento ST: Registro VICTIM
	Access to Reperfusion Therapy and Mortality in Women with ST-Segment–Elevation Myocardial Infarction: VICTIM Register
https://abccardiol.org/wp-content/uploads/articles_xml/1678-4170-abc-116-04-0795/1678-4170-abc-116-04-0795.pdf	Hussid et al.
	Obesidade Visceral e Hipertensão Sistólica como Substratos da Disfunção Endotelial em Adolescentes Obesos
	Visceral Obesity and High Systolic Blood Pressure as the Substrate of Endothelial Dysfunction in Obese Adolescents
https://abccardiol.org/wp-content/uploads/articles_xml/0066-782X-abc-116-01-0004/0066-782X-abc-116-01-0004.x44344.pdf	Santos et al.
	Treino de Força Reduz Stress Oxidativo Cardíaco e Renal em Ratos com Hipertensão Renovascular
	Strength Training Reduces Cardiac and Renal Oxidative Stress in Rats with Renovascular Hypertension
https://abccardiol.org/wp-content/uploads/articles_xml/0066-782X-abc-117-02-0309/0066-782X-abc-117-02-0309.x44344.pdf	Chehuen et al.
	Respostas Fisiológicas à Caminhada Máxima e Submáxima em Pacientes com Doença Arterial Periférica Sintomática
	Physiological Responses to Maximal and Submaximal Walking in Patients with Symptomatic Peripheral Artery Disease
https://abccardiol.org/wp-content/uploads/articles_xml/0066-782X-abc-116-02-0266/0066-782X-abc-116-02-0266.x27815.pdf	Guimarães et al.
	Aumento de Óbitos Domiciliares devido a Parada Cardiorrespiratória em Tempos de Pandemia de COVID-19
	Increased Home Death Due to Cardiopulmonary Arrest in Times of COVID-19 Pandemic

**Tabela 2:** Lista com a seleção dos dez melhores artigos publicados na *Revista Portuguesa de Cardiologia* em 2021

Autores	Título do artigo
Mesquita et al.	Cardiac arrhythmias in patients presenting with COVID-19 treated in Portuguese hospitals: A national registry from the Portuguese Association of Arrhythmology, Pacing and Electrophysiology
Manuel et al.	Long-term outcomes after radiofrequency catheter ablation of the atrioventricular node: The experience of a Portuguese tertiary center
Ribeiro et al.	Impact of catheter ablation for atrial fibrillation in patients with heart failure and left ventricular systolic dysfunction
Velásquez-Rodríguez et al.	Influence of left ventricular systolic function on the long-term benefit of beta-blockers after ST-segment elevation myocardial infarction
Raposo et al.	Adoption and patterns of use of invasive physiological assessment of coronary artery disease in a large cohort of 40 821 real-world procedures over a 12-year period
Costa et al.	Atherosclerosis: The cost of illness in Portugal
Silva et al.	Prognostic impact of iron deficiency in acute coronary syndromes
Paiva et al.	Non-vitamin K antagonist oral anticoagulation versus left atrial appendage occlusion for primary and secondary stroke prevention after cardioembolic stroke
Sousa et al.	The burden of infective endocarditis in Portugal in the last 30 years: a systematic review of observational studies
Mello Sampayo et al.	Cost-effectiveness of cardio-oncology clinical assessment for prevention of chemotherapy-induced cardiotoxicity

A seguir, apresentamos uma lista dos dez melhores artigos em cada revista e sua breve descrição, assim como as suas principais implicações para o diagnóstico, o tratamento e o entendimento da doença cardiovascular. Visando à melhor compreensão dos assuntos, os artigos foram agrupados e são apresentados de acordo com quatro temas gerais.

## COVID-19 e suas consequências

Os anos de 2020 e 2021 foram marcados pelo enorme impacto da pandemia de COVID-19 nos cuidados de saúde. Em estudo publicado na RPC, Mesquita et al.^4^ analisaram a prevalência e o impacto prognóstico das arritmias cardíacas em pacientes hospitalizados por COVID-19. Foi utilizado um registro da Associação Portuguesa de Arritmologia, *Pacing* e Eletrofisiologia com dados provenientes de 20 hospitais portugueses, relativos a 692 pacientes internados devido à COVID-19. Foi observado que, naquela população de pacientes, ocorreram eventos arrítmicos em 11,7%. As arritmias mais frequentes foram a fibrilação e o *flutter* atriais (FFA - 62,5%). Dois pacientes (3,1%) apresentaram taquicardia ventricular e 17 (26,6%) apresentaram taquicardia supraventricular paroxística. De forma surpreendente, nenhum desses pacientes teve complicações importantes decorrentes do evento arrítmico (ou morte de causa arrítmica), apesar de se tratar de um grupo com COVID-19 mais grave e que apresentava muitas comorbidades, e maior frequência de instabilidade hemodinâmica e/ou disfunção multiorgânica. Apesar de 76,6% dos pacientes com eventos arrítmicos estarem sob medicação que aumenta o intervalo QT (ritonavir/lopinavir, hidroxicloroquina ou azitromicina), apenas sete (10,9%) apresentaram um intervalo QTc prolongado (variando entre 480ms e 596 ms).^4^ Desse estudo pode-se concluir que a incidência de arritmias cardíacas é alta nos pacientes hospitalizados por COVID-19, mas elas não se associaram a aumento da mortalidade cardíaca, embora ocorram com frequência naqueles com doença mais grave e com disfunção multiorgânica. A incidência de arritmias ventriculares foi baixa apesar de os pacientes estarem medicados com terapêuticas que prolongam o intervalo QT.

Estudo observacional avaliou a taxa de mortalidade por parada cardiorrespiratória (PCR) em relação ao número total de atendimentos domiciliares notificados pelo Serviço de Atendimento Móvel de Urgência (SAMU) em Belo Horizonte, Minas Gerais, Brasil, em março de 2018, 2019 e 2020. Observou-se aumento numérico gradativo da taxa de óbitos domiciliares por PCR para o total de atendimentos pelo SAMU e aumento proporcional de 33% dos óbitos domiciliares em março de 2020, mês do início da pandemia de COVID-19. A maioria dos pacientes (63,8%) tinha 60 anos ou mais e cerca de 87% dos casos de PCR notificados foram associados com comorbidades clínicas, como hipertensão arterial sistêmica (22,87%), insuficiência cardíaca (13,03%) e diabetes mellitus (11,0%). Cabe ressaltar que, embora os familiares sinalizassem a presença de comorbidades nos pacientes, em 38,4% dos casos relatados, os familiares não souberam informar quais eram essas comorbidades.^5^ É importante que o sistema de saúde brasileiro aprimore o conhecimento dos pacientes e familiares sobre as doenças com as quais convivem, salientando e facilitando o acesso ao sistema hospitalar, para diminuir o impacto das PCR extra-hospitalares, que apresentam pequenas chances de sobrevida.

Fernandes et al.^6^ realizaram um estudo retrospectivo de 187 pacientes admitidos numa unidade de terapia intensiva (UTI) na sequência de PCR ao longo de um período de 5 anos. A idade mediana dos pacientes foi de 67 anos. A PCR foi intra-hospitalar em 61% dos casos, tendo sido o ritmo inicial de PCR não desfibrilável em 87%. O tempo médio até retorno da circulação espontânea (ROSC) foi de 10 minutos. A PCR foi de presumível causa cardíaca em apenas 31% dos casos, o que é explicado pela exclusão dos pacientes com PCR por infarto do miocárdio (IM) com supra de ST (CSST), pois eles foram admitidos diretamente na UTI coronária do mesmo hospital. Os autores reportam mortalidade intra-hospitalar de 63% (45% da qual em relação à suspensão de medidas de suporte orgânico) e mortalidade em um ano de 72%. A prevalência de alta com bom *status* neurológico (categoria de *performance* cerebral – CPC – de 1) foi de apenas 25%. Foram preditores independentes de mortalidade intra-hospitalar o tempo até início de suporte básico de vida (SBV), score SAPS II elevado, ritmo inicial não desfibrilável e tempo sob suporte de vasopressor. De forma surpreendente, apesar de o tempo até início de SBV e o tempo até ROSC terem sido superiores na população de pacientes com PCR extra-hospitalar, os resultados clínicos não diferiram significativamente nas duas populações de pacientes. A sobrevida com bom *status* neurológico (CPC 1 ou 2) esteve associada a atividade epilética menos frequente e suporte ventilatório por período de tempo mais curto, mas não se associou a PCR presenciada, ritmo inicial, tempo até ROSC, ou utilização de protocolo de normotermia. Por último, os desfechos neurológicos e de mortalidade foram semelhantes em ambos os sexos. Esse estudo salienta uma vez mais a importância de melhorar todos os componentes da cadeia de sobrevivência, no sentido de otimizar o prognóstico desses pacientes. Tem ainda o mérito de expor a necessidade premente de ensaios clínicos nessa área, idealmente multicêntricos e devidamente enquadrados do ponto de vista ético.^6^

### Arritmia cardíaca e seu impacto na sociedade

Fibrilação e *flutter* atriais são as arritmias mais comuns, tanto na população em geral, quanto nos pacientes com COVID-19, ainda que não tenham um caráter de associação com a presença de PCR.^4–7^ Com o objetivo de estudar a incidência e os fatores associados às doenças cardiovasculares e ao diabetes, foi realizado o Estudo Longitudinal de Saúde do Adulto (ELSA-Brasil), que é uma grande coorte multicêntrica de indivíduos entre 35 e 74 anos, provenientes de seis cidades brasileiras. Um subestudo^8^ do ELSA-Brasil, com os 13.260 participantes, investigou a presença de FFA, que foram definidos pelo eletrocardiograma ou por autorrelato. Teve como objetivo analisar se a idade e o sexo estavam associados com o uso de anticoagulantes para evitar acidente vascular cerebral (AVC). Os autores observaram que a maioria dos participantes era do sexo feminino (54,4%), com idade mediana de 51 anos, sendo FFA detectados em 333 (2,5%) participantes. Os autores observaram que idade (RC: 1,05; IC95%: 1,04-1,07), hipertensão arterial (RC: 1,44; IC95%: 1,14-1,81), doença arterial coronariana (DAC -RC: 5,11; IC95%: 3,85-6,79), insuficiência cardíaca (RC: 7,37; IC95%: 5,00-10,87) e febre reumática (RC: 3,38; IC95%: 2,28-5,02) foram associadas com FFA. Apenas 20 pacientes com pontuação no CHA_2_DS_2_-VASc ≥2 (10,8%) usavam anticoagulantes, sendo esse uso menor em mulheres. Esses achados representam um grande desafio para os cuidados de saúde de FFA.

Em 2018, o estudo CASTLE-AF^9^ mostrou que a ablação da fibrilação atrial (FA) numa população selecionada de pacientes com insuficiência cardíaca poderia melhorar seu prognóstico. Em outro estudo, Ribeiro et al.^10^ avaliaram retrospectivamente o impacto da ablação da FA em 22 pacientes com insuficiência cardíaca (32% em classe NYHA II e 58% em classe NYHA III) e fração de ejeção ventricular esquerda (FEVE) <50%. O procedimento foi realizado com sucesso em 100% dos pacientes, sem complicações registradas. A recorrência de FA após o período de *blanking* foi de 18%. Após um tempo de seguimento mediano de 11 meses, os autores reportaram uma melhora da capacidade funcional, com classe funcional da NYHA média de 2,35±0,49 antes do procedimento e de 1,3±0,47 após o procedimento (p<0,001). Houve ainda melhoria da FEVE média de 40% para 58% (p<0,01) e remodelamento favorável das câmaras cardíacas esquerdas. Os autores concluíram que, em pacientes cuidadosamente selecionados com FA e insuficiência cardíaca com FEVE <50%, a ablação de FA resulta em melhora significativa da classe funcional, melhora da FEVE e em remodelamento estrutural favorável das câmaras cardíacas esquerdas. Propõem assim que, tal como proposto por ensaios clínicos de larga escala recentemente publicados,^11^ a ablação de FA seja considerada precocemente nesses pacientes, atendendo ao elevado perfil de segurança quando comparado com estratégias farmacológicas de controle de ritmo.

Na área da eletrofisiologia invasiva, Manuel et al.^12^ publicaram um interessante estudo com o seguimento a longo prazo de pacientes submetidos a ablação do nó atrioventricular com radiofrequência. Foram analisados os dados de 123 pacientes (idade média de 69±9 anos) de um centro terciário português, com seguimento mediano de 8,5 anos. As indicações para a realização desse procedimento foram a baixa porcentagem de *pacing* biventricular (em 8%), a presença de taquicardiomiopatia (em 80%, dos quais 65% eram relativos a FA de resposta ventricular rápida apesar de terapêutica farmacológica), a ocorrência de choques inapropriados de cardiodesfibrilador implantável (em 2%) ou taquicardia supraventricular incontrolável (em 10%). Em 89% dos casos, foi implantado um dispositivo no momento da ablação (14% dos quais foram dispositivos de ressincronização cardíaca) e, nos restantes, os pacientes já eram portadores desses dispositivos. O procedimento foi realizado com sucesso em todos os pacientes, sem complicações maiores periprocedimento. De forma notável, durante esse período de *follow-up,* não foram documentadas complicações maiores associadas aos dispositivos cardíacos implantáveis. Os autores reportam ainda melhoria da classe funcional da NYHA, menos hospitalizações e uma redução das visitas não planejadas por descompensação de insuficiência cardíaca. A mortalidade por todas as causas foi de 3,5% no final do primeiro ano e de 23% ao longo de todo o seguimento, em linha com o descrito na literatura. Esse estudo mostra o perfil de elevada eficácia e segurança do procedimento de ablação do nó atrioventricular em pacientes selecionados, uma técnica que pode ser especialmente importante no tratamento de população complexa de pacientes, podendo permitir a sua melhoria sintomática e a redução de hospitalização por insuficiência cardíaca, conforme referido nas diretrizes europeias.^13^

A oclusão percutânea do apêndice atrial esquerdo (OPAAE) é um tema controverso da cardiologia atual. Em estudo publicado na RPC, Paiva et al.^14^ analisaram a segurança e a eficácia desse procedimento em pacientes com FA “não valvular”, quer em prevenção primária quer secundária de AVC. Os autores realizaram um estudo observacional prospetivo envolvendo 91 pacientes submetidos a OPAAE e 149 pacientes tratados com os anticoagulantes orais diretos (DOAC), ao longo de um seguimento médio de 13 meses. Os pacientes submetidos a OPAAE tinham idade média de 74,7±8,7 anos (vs. 77,8±8,0 anos dos pacientes em uso de DOAC), 59% eram do sexo masculino, com CHA_2_DS_2_-VASc escore médio de 4,3±1,4 (vs. 5,3±1,3) e HAS-BLED escore médio de 3,0±0,9 (vs. 4,0±0,7). Foram utilizados os dispositivos ACP/Amulet™ e Watchman™ e a taxa de sucesso do procedimento foi de 96,3%, sem complicações maiores registradas. O estudo demonstrou uma tendência não significativa para redução do desfecho composto de morte, AVC ou hemorragia maior nos pacientes submetidos a OPAAE (11,0% vs. 20,9%; HR: 0,42, IC95%: 0,17-1,05, p=0,06). Em cerca de 20% dos pacientes submetidos a OPAAE que suspenderam a terapêutica antiplaquetária após seis meses do implante do dispositivo por hemorragia menor, não houve excesso de eventos cardiovasculares ou hemorragias graves. Os autores concluem que a OPAAE não foi inferior aos DOAC para prevenção do desfecho primário composto por morte, AVC e hemorragia maior numa população de pacientes com FA “não valvular”. A comunidade científica aguarda expectante pelo resultado dos diversos ensaios clínicos randomizados em curso nessa área, que poderão fornecer respostas mais definitivas quanto ao papel da OPAAE no tratamento de populações de alto risco (por exemplo, pacientes com história de hemorragia intracraniana).^15–18^

O maior questionamento acerca da OPAAE é sua custo-efetividade, ainda não respondida na literatura disponível. De fato, a economia da saúde tornou-se um dos temas mais relevantes atualmente, em especial em países como o Brasil que oferecem acesso universal ao sistema de saúde. Um bom exemplo é o custo associado com a aterosclerose que se constitui no denominador patológico comum às principais causas de morbimortalidade dos países desenvolvidos, incluindo, entre outras, as síndromes coronarianas agudas e crônicas, os AVC isquêmicos e a doença arterial periférica. Em artigo publicado na RPC, Costa et al.^19^ procuraram quantificar o impacto econômico da aterosclerose em Portugal, usando para isso dados de prevalência e recorrendo a múltiplas bases de dados nacionais. Os custos da aterosclerose totalizaram 1,9 mil milhões de euros por ano, o que corresponde a 11% de todos os gastos em saúde e a aproximadamente 1% do PIB (dados de 2016). Desses custos, 58% representaram custos diretos com a doença (55% dos quais relacionados com cuidados de saúde primários) e 42% foram custos indiretos (91% dos quais por absenteísmo laboral). Entre as manifestações da aterosclerose, a doença cardíaca isquêmica apresentou maior custo por paciente, que se deveu sobretudo ao custo com a medicação. É possível concluir que, atendendo à sua elevada prevalência (cerca de 9% da população adulta em Portugal) e impacto econômico, a aterosclerose constitui ainda um desafio clínico, social e financeiro para os sistemas de saúde em todo o mundo, o que poderá ser agravado num futuro próximo, devido ao envelhecimento da população.

### Desafios atuais na doença arterial coronariana

Como mencionado, o maior custo das doenças ateroscleróticas, por paciente, relaciona-se com a doença cardíaca isquêmica e sua abordagem, notadamente devido às diferentes terapêuticas disponíveis.^19^ Vários estudos mostraram que a avaliação funcional invasiva coronária com reserva fracionada de fluxo miocárdico (do inglês *fractional flow reserve*, FFR ou iFFR) pode ser custo-efetiva^20^ e melhorar o prognóstico.^21–23^ Contudo, a sua utilização na prática clínica é ainda residual na maioria dos laboratórios de cardiologia de intervenção. Num interessante trabalho publicado, Raposo et al.^24^ analisaram o padrão de utilização dessas técnicas de avaliação funcional invasiva de DAC em dois centros de referência ao longo de um período de 12 anos, totalizando 40.821 pacientes submetidos a coronariografia invasiva. Essas técnicas foram utilizadas em apenas 0,6% dos casos submetidos a coronariografia por doença valvar e em 6,0% dos casos submetidos a coronariografia por DAC estável. Em 42,9% dos pacientes com DAC estável submetidos a angioplastia, não havia documentação prévia de isquemia por testes de imagem, nem foi utilizada fisiologia invasiva. A idade dos operadores e a hora de realização do procedimento associaram-se de forma significativa à utilização desses índices. O *timing* de publicação de ensaios clínicos de referência e de recomendações internacionais relevantes relacionou-se com a maior taxa de adoção dessas técnicas. A emergência de evidência científica quanto aos índices não hiperêmicos (i.e., iFFR), de maior facilidade e rapidez de utilização, aumentou a proporção de sua utilização relativamente aos índices hiperêmicos (i.e., FFR), sem, no entanto, aumentar a taxa global de utilização. Os autores concluem que a taxa de utilização subótima da avaliação funcional invasiva de DAC constitui uma oportunidade para, através de estratégias dedicadas que visem aumentar a aderência às recomendações científicas e diminuir a inércia clínica, melhorar o prognóstico dos pacientes com DAC angiograficamente moderada.

A quantificação não invasiva da reserva fracionada de fluxo miocárdico pode ser particularmente útil nas estenoses moderadas (50% a 69%), auxiliando na discriminação das lesões associadas com isquemia significativa.^25^ Estudos recentes demonstraram que a angiotomografia de coronárias tem elevada acurácia para identificar isquemia miocárdica por meio da quantificação não invasiva da FFR (FFR_TC_) quando comparada a FFR ou iFFR, considerada padrão-ouro.^26,27^ Interessante estudo retrospectivo com pacientes encaminhados a angiotomografia de artérias coronárias e angiografia coronariana avaliou o desempenho diagnóstico da FFR_TC_ na detecção de DAC significativa em relação à FFR, definindo DAC obstrutiva como angiotomografia com redução luminal ≥50% e DAC funcionalmente obstrutiva como FFR ≤0,8. O estudo incluiu 93 pacientes consecutivos (152 vasos) e analisou a FFR_TC_ através de *software* baseado em aprendizado de máquina. Foi observada boa concordância em relação à medida de FFR, com tempo de pós-processamento em torno de 10 minutos. Com relação à *performance* diagnóstica, mesmo em tomógrafos de gerações anteriores, houve boa concordância entre FFR_TC_ e FFR, com mínima superestimação da FFR_TC_ (viés: -0,02; limites de concordância: 0,14 a 0,09). A FFR_TC_ demonstrou *performance* significativamente superior à classificação visual de estenose coronariana, reduzindo o número de casos falso-positivos. Os autores concluíram que a FFR_TC_ baseada em inteligência artificial, mesmo em tomógrafos de 128 e 256 cortes, apresenta boa *performance* diagnóstica na detecção de DAC, podendo reduzir procedimentos invasivos.^28^

De acordo com dados do Sistema Único de Saúde Brasileiro (SUS), o número de hospitalizações por IM no sistema público aumentou 54% de 2008 a 2019, ajustado para a população. Os procedimentos de intervenção coronariana percutânea (ICP) não primária por habitante dobraram, enquanto os de ICP primária aumentaram 31%. A taxa de mortalidade hospitalar por IM diminuiu de 15,9% em 2008 para 12,9% em 2019, sendo ainda bastante elevada se comparada com as taxas mundiais.^7^ Cabe ressaltar que o acesso a reperfusão miocárdica, que é o pilar terapêutico do IM, não está disponível para todos, especialmente para as mulheres, gerando maior mortalidade e impactando os custos totais da doença aterotrombótica. Estudo transversal com dados do Registro VICTIM avaliou pacientes com diagnóstico de IMCSST oriundos de quatro hospitais (um público e três privados) com disponibilidade para realizar angioplastia primária, em Sergipe, de dezembro de 2014 a junho de 2018. Foram incluídos 878 pacientes com IMCSST, sendo que 33,4% eram mulheres. Pouco mais da metade dos pacientes foi submetida à reperfusão miocárdica (53,3%, 134 mulheres e 334 homens). As mulheres apresentaram menores taxas de fibrinólise (2,3% no total, 1,7% nas mulheres e 2,6% nos homens) e de angioplastia primária (44% nas mulheres e 54,5% nos homens), com consequente maior mortalidade hospitalar nas mulheres do que nos homens (16,1% vs. 6,7%). Cabe ressaltar que as mulheres tinham maiores taxas de diabetes mellitus (42% vs. 28,5%), hipertensão arterial sistêmica (75,1% vs. 59%) e dislipidemia (50,2% vs. 33,3%) do que os homens. Os autores reforçam o achado de piores resultados para os usuários do serviço público, principalmente entre as mulheres, e apontam a necessidade de melhorias no acesso das mulheres portadoras de IMCSST a estratégias eficazes de tratamento, com o objetivo de reduzir a mortalidade hospitalar.^29^

A angioplastia primária tem como objetivo a recuperação da patência do lúmen arterial para promover o fluxo sanguíneo na microcirculação coronária. Contudo, um em cada três pacientes permanece com o fluxo microvascular reduzido mesmo com fluxo macrovascular restaurado, fenômeno conhecido como *no-reflow*. Esses pacientes têm maior risco de insuficiência cardíaca, choque cardiogênico e morte cardiovascular.^30^ Ainda que o escore SYNTAX seja bom preditor de disfunção microvascular, as cargas aterosclerótica e trombótica não são consideradas no algoritmo, devido à exclusão de lesões obstrutivas com estenoses menores que 50% e à atribuição de pontuação baixa para a presença ou ausência de trombo, respectivamente. Para avaliar se a carga aterosclerótica (através do escore Gensini) e a carga trombótica na artéria coronária culpada melhorariam a capacidade do escore SYNTAX para detectar *no-reflow*, Matos et al.^31^ incluíram 481 pacientes consecutivos portadores de IMCSST, com delta t de até 12 horas. Definiram *no-reflow* como fluxo TIMI < 3 ou fluxo TIMI =3, mas grau de *blush* miocárdico (*myocardial blush grade*) < 2, sendo a carga trombótica quantificada de acordo com o grau TIMI de trombo (0 a 5). A média da idade foi de 61±11 anos e o fenômeno de *no-reflow* ocorreu em 32,8% dos pacientes. Os preditores independentes de *no-reflow* foram escore SYNTAX (OR=1,05, IC95%: 1,01–1,08, p<0,01), carga trombótica (OR=1,17, IC95%: 1,06–1,31, p<0,01) e escore Gensini (OR=1,37, IC95%: 1,13–1,65, p<0,01). Os escores combinados apresentaram uma maior área sob a curva quando comparados ao escore SYNTAX isolado (0,78 [0,73–0,82] vs. 0,73 [0,68–0,78], p=0,03). Os autores concluíram que as cargas aterosclerótica e trombótica na artéria culpada adicionam valor preditivo ao escore SYNTAX na detecção do fenômeno *no-reflow*. A maior limitação do estudo é ter caráter transversal e ser unicêntrico.

Uma das estratégias para melhorar a mortalidade por IM seria o uso de betabloqueadores (BB) e ácido acetilsalicílico. Recentemente têm-se questionado se os BB terão benefício prognóstico a longo prazo após síndrome coronariana aguda. Em estudo retrospectivo publicado na RPC, Velásquez-Rodríguez et al.^32^ procuraram responder a essa questão analisando 972 pacientes consecutivos admitidos com IMCSST, 99,7% dos quais submetidos a ICP, com idade média de 62,6±13,5 anos, sendo 21,8% do sexo feminino. À data de alta, 85,9% dos pacientes foram medicados com BB. Tal como esperado, observou-se que a população de pacientes não medicados com BB apresentava tendencialmente mais comorbidades (como neoplasia, anemia, doença pulmonar obstrutiva crônica), maior prevalência de IM em território inferior, bem como de bloqueio atrioventricular de alto grau. Após um período de *follow-up* médio de 49,6±24,9 meses, o uso de BB foi preditor independente de sobrevida na população geral (HR 0,61, IC95%: 0,38-0,96), mas, quando estratificada pelo valor de FEVE, apenas a população com FEVE ≤40% pareceu obter benefício de sobrevida com essa terapêutica. Os autores concluíram que os resultados do estudo levantam dúvidas razoáveis quanto ao real benefício do uso sistemático de BB a longo prazo, após IMCSST, em pacientes com FEVE superior a 40%. Em outro estudo observacional incluindo 1520 pacientes, publicado na RPC em 2018, Timóteo et al.^33^ concluíram pelo benefício no uso sistemático de BB após síndrome coronariana aguda, independentemente do valor da FEVE. Em suma, parece razoável afirmar em 2022 que, na ausência de evidência científica proveniente de ensaios clínicos randomizados adequadamente dimensionados, há ainda lugar para uma individualização de terapêutica com BB após IM nos pacientes com FEVE >40% que leve em conta a esperança média de vida do paciente, suas preferências, estado funcional e cognitivo, perfil de comorbidades, fragilidade, interações medicamentosas, reações adversas, entre outros.^34^

Novas estratégias para minimizar os desfechos adversos decorrentes do IM são desejadas, procurando-se extrapolar os dados dos pacientes com insuficiência cardíaca de etiologia isquêmica.^35^ Existem poucos dados na literatura relativamente ao impacto prognóstico da deficiência de ferro nas síndromes coronarianas agudas. Estudo publicado na RPC por Silva et al.^36^ procurou responder a essa lacuna. Foram avaliados os dados de 817 pacientes internados por síndrome coronariana aguda num hospital terciário português, que foram alocados em dois grupos de acordo com a presença (n=298, 36%) ou ausência (n=519) de deficiência de ferro na admissão. Os pacientes com deficiência de ferro apresentaram mais frequentemente depressão moderada e grave da função ventricular esquerda, depressão da função do ventrículo direito e classes Killip mais elevadas. No seguimento a médio prazo (média de 738 dias), esses pacientes apresentaram também maior mortalidade por qualquer causa (12,6% vs. 6,3%, p=0,04), insuficiência cardíaca NYHA III/IV (10,5% vs. 5,3%, p=0,011), bem como maior taxa de readmissão hospitalar (9,8% vs. 13,7%, p=0,048). A deficiência de ferro constituiu-se um preditor independente de morte ou insuficiência cardíaca no *follow-up* e possibilitou ainda a estratificação prognóstica dos pacientes sem anemia e/ou com classes Killip mais baixas (≤2) em termos de ocorrência de morte ou insuficiência cardíaca. Os autores concluíram que a deficiência de ferro é uma condição prevalente nos pacientes com síndromes coronarianas agudas, sendo um fator preditor independente de morte ou insuficiência cardíaca grave no *follow-up* a médio prazo. Para além disso, a deficiência de ferro pode ser um interessante complemento na estratificação prognóstica dos pacientes com síndromes coronarianas agudas sem anemia, bem como naqueles com classes Killip ≤2.

### Insuficiência cardíaca: das causas à prevenção

A insuficiência cardíaca afeta aproximadamente 26 milhões de pessoas em todo o mundo e esses números tendem a aumentar com o envelhecimento populacional, com a alta prevalência de fatores de risco cardiovascular, com a sobrevivência dos pacientes ao IM e com as melhorias terapêuticas da insuficiência cardíaca. Para além disso, parece haver associação entre piores desfechos da insuficiência cardíaca e os determinantes sociais.^37^ Santos et al.^38^ analisaram a evolução temporal das taxas de mortalidade por insuficiência cardíaca por sexo e faixa etária no Brasil, regiões geográficas (RG) e unidades da federação (UF), de 1980 a 2018, e associações com o Índice de Desenvolvimento Humano Municipal (IDHM). A mortalidade por insuficiência cardíaca diminuiu no Brasil ao longo dos 29 anos estudados, apresentando tendência de redução progressiva a partir de 2008, atingindo ao final de 2018 patamar semelhante nas RG e UF. As taxas de mortalidade por insuficiência cardíaca no sexo masculino foram maiores durante quase todos os períodos e faixas etárias observadas, provavelmente por relação com a etiologia isquêmica da insuficiência cardíaca. Observou-se tendência inversa entre a variação da taxa de mortalidade das UF entre 1990 e 2018 e a variação do respectivo IDHM entre 1991 e 2010. Os autores sugeriram que, em relação à mortalidade por insuficiência cardíaca, mais importante que o grau de incremento do IDHM é o nível final que ele alcança, IDHM igual ou superior a 0,7. Os autores concluem que esforços devem ser feitos no sentido de ampliar o acesso à assistência à saúde e o controle mais efetivo dos fatores de risco cardiovascular, dislipidemia, obesidade, sedentarismo, diabetes, bem como dos determinantes sociais, que contribuem para a mortalidade tanto por doença isquêmica do coração quanto por insuficiência cardíaca.

Tendo em mente a crescente população em risco de desenvolver insuficiência cardíaca na sequência de exposição a quimioterapia (QT) cardiotóxica, bem como a escassez de evidência científica para suportar diferentes estratégias de cardioproteção nesses pacientes, Mello et al.^39^ publicaram um estudo que tenta nos dar algumas respostas, partindo de uma lógica de custo-efetividade. Para esse efeito, foram calculados e comparados os QALYs de duas estratégias de cardioproteção: uma guiada por vigilância imagiológica da FEVE (em que a medicação cardioprotetora era prescrita na ocorrência de insuficiência cardíaca sob QT, definida pela queda sintomática de >10% na FEVE, para um valor final ≤55%) e outra estratégia de “cardioproteção universal” (todos os pacientes medicados com BB e inibidor da enzima de conversão da angiotensina). Para esses cálculos foi usada uma simulação de Monte Carlo de um modelo de Markov. Os autores concluíram que, em uma perspectiva de custo-efetividade, a estratégia de cardioproteção guiada por vigilância imagiológica era superior à estratégia de cardioproteção universal, permitindo a obtenção de mais QALYs a um custo inferior. Num caso de referência de uma mulher de 63 anos com neoplasia de mama submetida a QT com antraciclinas e trastuzumabe ao longo de 5 anos, a primeira estratégia resultou em 4,22 QALYs a um custo de €2594 e a segunda estratégia resultou em 3,42 QALYs a um custo de €3758. São definitivamente necessários ensaios clínicos de larga escala para uma melhor definição da população de pacientes que se beneficia de estratégias de cardioproteção em “prevenção primária” de cardiotoxicidade.

Também considerados como preditores de insuficiência cardíaca, os distúrbios relacionados à obesidade, por exemplo resistência à insulina, diabetes e dislipidemia, associam-se à disfunção do tecido adiposo, promovendo respostas desadaptativas no coração, como hipertrofia de miócitos, disfunção contrátil e remodelação cardíaca, que contribuem para o desenvolvimento de ambos e para a progressão da insuficiência cardíaca crônica.^40^ Alves et al.,^41^ em elegante estudo com ratos Wistar, levantaram a hipótese de que a ativação do receptor TLR-4 (do inglês, *toll-like receptor 4*) participa da doença cardíaca relacionada à obesidade por desencadear a produção de citocinas via NF-ĸB (do inglês, *nuclear factor-*ĸ*B*). O grupo “obeso”, que foi alimentado com dieta rica em açúcar e gordura e água mais 25% de sacarose por 30 semanas, apresentou obesidade, elevação dos níveis de glicose, triglicerídeos e ácido úrico, resistência à insulina, além de elevação da pressão arterial sistólica e do fator de necrose tumoral alfa (TNF-α) no tecido adiposo. Apresentou também remodelação cardíaca e disfunção diastólica. A expressão de TLR-4 e NF-ĸB e os níveis de citocinas foram maiores no grupo de ratos Wistar obesos. Nesse grupo houve maior expressão do gene e da proteína TLR-4 juntamente com aumento da fosforilação do NF-ĸB, confirmando a ativação dessa via como mediadora da inflamação. Os autores concluem que a resposta imune inata por meio da ativação do receptor TLR-4 é um dos mecanismos que pode contribuir para o surgimento do processo inflamatório miocárdico na obesidade e que a inflamação derivada da ativação do TLR-4 cardíaco é um novo mecanismo que pode levar à remodelação e disfunção cardíaca.

A obesidade na adolescência pode levar a síndrome metabólica (SM) e disfunção endotelial, sendo preditor de obesidade na fase adulta, além de marcador precoce de risco cardiovascular. Importante notar ainda que as doenças respiratórias do sono estão entre as consequências da obesidade, incluindo a síndrome da apneia obstrutiva do sono (SAOS).^42^ Com o objetivo de investigar se a obesidade durante a adolescência está associada com SM e/ou SAOS, além de sua relação com a disfunção endotelial, Hussid et al.^43^ estudaram 20 adolescentes obesos sedentários (14,2±1,6 anos, 100,9±20,3 kg) e 10 adolescentes eutróficos (15,2±1,2 anos, 54,4±5,3 kg) pareados por sexo. Os autores avaliaram os fatores de risco para SM, função vascular (dilatação mediada pelo fluxo), capacidade funcional (VO_2_pico) e presença de SAOS (índice de apneia-hipopneia > 1 evento/hora, pela polissonografia). Na amostra estudada, a obesidade foi um importante fator de risco para o desenvolvimento de SM e levou à disfunção endotelial. A circunferência da cintura e a pressão arterial sistólica aumentadas foram preditoras de disfunção endotelial em adolescentes. A SAOS estava presente na maioria dos adolescentes, independentemente da obesidade. O tamanho amostral reduzido, o período do ciclo menstrual nas meninas com a atuação dos hormônios na função endotelial e a ausência de padrões para as variáveis estudadas entre os adolescentes brasileiros podem ter influenciado os achados desse estudo.^43^

O transplante cardíaco (TC) é o padrão-ouro do cuidado da insuficiência cardíaca terminal e existe forte associação entre pressão arterial pulmonar sistólica (PAPs) e letalidade por esse procedimento, sendo a hipertensão pulmonar (HP) grave uma das contraindicações maiores para o transplante devido à disfunção do coração direito pós-operatória.^44^ Mendes et al.^45^ estudaram 300 candidatos a TC consecutivos tratados entre 2003 e 2013, dividindo-os em dois grupos de acordo com a presença de HP. Desses, 95 pacientes tinham HP fixa e, dentre eles, 30 foram tratados com sildenafil e foram para o TC, formando o grupo A. O grupo B incluiu 205 pacientes sem HP que também foram submetidos ao TC. A PAPs diminuiu após o TC em ambos os grupos, mas permaneceu significativamente alta no grupo A em relação ao grupo B (40,3 ± 8,0 mmHg vs. 36,5 ± 11,5 mmHg, p=0,022). Um ano após o TC, a PAPs era 32,4 ± 6,3 mmHg no grupo A versus 30,5 ± 8,2 mmHg no grupo B (p=0,274). Os autores concluíram que, nos pacientes com HP pré-tratados com sildenafil, a hemodinâmica pós-operatória inicial e o prognóstico são numericamente piores quando comparados com os de pacientes sem HP; contudo, depois de 1 ano, as mortalidades em médio e longo prazo se assemelham. Salientam que o uso do sildenafil para reduzir a PAPs pode ser considerado uma “terapia de resgate” valiosa em um grupo de pacientes com insuficiência cardíaca terminal.

Os exercícios físicos minimizam os maiores determinantes da insuficiência cardíaca, como obesidade, hipertensão e isquemia miocárdica e auxiliam tanto na prevenção primária, quanto secundária da doença aterotrombótica. Os pacientes com doença arterial periférica (DAP) sintomática, notadamente com claudicação intermitente, apresentam hipertensão arterial, disfunção autonômica cardíaca, disfunção endotelial, aumento do estresse oxidativo e inflamação. A DAP é ainda um marcador de carga aterosclerótica, associando-se com doença aterotrombótica ‘multissítio’. O treinamento de exercício é considerado o melhor tratamento para pacientes com DAP sintomática por melhorar a capacidade de locomoção, os sintomas de claudicação, a qualidade de vida e a saúde cardiovascular desses pacientes.^46^ Chehuen et al.^47^ realizaram um estudo para comparar os efeitos agudos de caminhada máxima e submáxima na função cardiovascular e avaliar a regulação e os processos fisiopatológicos associados pós-exercício em pacientes com DAP sintomática. Os autores recrutaram 30 homens que foram submetidos a duas sessões: caminhada máxima (protocolo de Gardner) e caminhada submáxima (15 períodos de 2 minutos de caminhada separados por 2 minutos de repouso ereto). Em cada sessão, avaliaram antes e após a caminhada: os sinais vitais, a variabilidade da frequência cardíaca, os fluxos sanguíneos do antebraço e da panturrilha, a hiperemia reativa, a peroxidação lipídica, e as concentrações plasmáticas de óxido nítrico, proteína C reativa, TNF-α e moléculas de adesão vascular e intercelular (VCAM e ICAM, respectivamente). Observaram que a caminhada submáxima, mas não a máxima, reduziu a pressão arterial pós-exercício, enquanto a caminhada máxima manteve a sobrecarga cardíaca elevada durante o período de recuperação. As sessões de caminhada máxima e submáxima aumentaram a frequência cardíaca, o equilíbrio simpatovagal cardíaco e a inflamação pós-exercício de forma semelhante, enquanto não alteraram a biodisponibilidade de óxido nítrico e o estresse oxidativo pós-exercício. Novos estudos longitudinais com populações maiores e diversas precisarão ser realizados para mensurar os efeitos no longo prazo e estabelecer a melhor abordagem para os pacientes com DAP sintomática.

Ainda em relação ao exercício físico, Santos et al.^48^ estudaram ratos Wistar machos, com idade de 10 a 12 semanas, para verificar se o treino de força reduziria o dano oxidativo ao coração e ao rim contralateral causado por cirurgia de indução de hipertensão renovascular. Os autores avaliaram as alterações na atividade das enzimas antioxidantes endógenas superóxido dismutase, catalase e glutationa peroxidase. O treino de força foi iniciado quatro semanas após a indução da hipertensão renovascular, teve 12 semanas de duração e foi realizado a 70% de 1RM. Após o treino de força, houve redução de danos oxidativos a lipídios e proteínas, com redução de peróxidos de hidrogênio e níveis sulfidrílicos totais, respectivamente, e aumento nas atividades das enzimas antioxidantes superóxido dismutase, catalase e glutationa peroxidase. Os autores concluem que o treino de força tem o potencial de reduzir danos oxidativos, aumentando a atividade de enzimas antioxidantes, e sugerem que o treino de força possa ser uma ferramenta não farmacológica para o tratamento de hipertensão renovascular, com o potencial de evitar o avanço dos danos ao coração e ao rim sem estenose arterial renal. Estudos em seres humanos precisarão ser realizados para a confirmação dessa hipótese.

Sousa et al.^49^ publicaram um estudo acerca da epidemiologia da endocardite infecciosa em Portugal. Trata-se de uma revisão sistemática de estudos observacionais, incluindo os dados de 1.872 pacientes, que possibilitou a avaliação de importantes tendências no diagnóstico, tratamento e prognóstico desses pacientes ao longo das últimas três décadas. A idade média foi de 55,5 ± 12,1 anos, com uma grande prevalência do sexo masculino. Ao longo do tempo, as séries reportaram uma tendência para um aumento na idade média dos pacientes, que se situava nos 61,6 ± 16,3 anos em 2008. A porcentagem de pacientes com endocardite de válvula protética ou de dispositivo cardíaco implantável foi também crescente nas últimas décadas, cifrando-se, respectivamente, em 22,6% e 6,0% nas séries mais contemporâneas. A prevalência de infeção por enterococo foi de 10,2%, em linha com os dados do registro europeu EURO-ENDO^50^ e refletindo uma vez mais o envelhecimento crescente dessa população de pacientes. O uso de técnicas de imagem cardíaca não ultrassonográfica (isto é, PET-FDG e angiotomografia computadorizada cardíaca) foi globalmente baixo, explicando-se pelo fato de a grande maioria dos pacientes ter sido tratada antes das recomendações europeias de 2015,^51^ em que a avaliação por essas técnicas integrou pela primeira vez um papel decisivo no diagnóstico e estratificação prognóstica. A taxa de cirurgia cardíaca foi muito variável (3,1% a 52%) e tendencialmente maior nas séries mais recentes. A mortalidade de curto prazo situou-se entre 3,0% e 37,2%. Esse estudo tem o mérito de fornecer informação epidemiológica importante acerca de uma patologia de prevalência e complexidade crescentes. Essa informação pode alavancar ensaios clínicos nessa área, em que a evidência científica é ainda escassa no que concerne à melhor estratégia diagnóstica e terapêutica.

## Conclusões

A despeito da pandemia de COVID-19, 2021 foi mais um ano de intensa atividade para as publicações científicas *Arquivos Brasileiros de Cardiologia* e *Revista Portuguesa de Cardiologia*. Nesta breve revisão dos melhores artigos do ano 2021, procuramos de forma clara e prática revisar achados desses artigos e colocá-los em um contexto que permita a compreensão do seu alcance clínico e do entendimento das doenças cardiovasculares. Entender como avançamos no ano passado é de fundamental importância para progredirmos ainda mais neste e nos próximos anos.

Os destaques de 2021 foram para áreas temáticas que incluem: COVID-19 e suas consequências, arritmia cardíaca e seu impacto na sociedade, desafios atuais na doença arterial coronária e insuficiência cardíaca: das causas à prevenção. Os artigos abordaram novas tecnologias, epidemiologia das doenças, estudos experimentais para entendimento fisiopatológico e combate a fatores de risco através do estilo de vida saudável, em particular com estudos abordando o exercício físico e seu papel nas doenças cardiovasculares.

Foi uma verdadeira honra e deleite para os autores desta seleção poder discorrer sobre artigos de tão alta qualidade no papel de editores das duas publicações científicas cardiovasculares mais importantes em língua portuguesa. Agradecemos muito a todos que continuam submetendo sua melhor ciência nas nossas publicações científicas. Esperamos que os leitores apreciem esta revisão e se sintam estimulados a terem seus próprios artigos na versão dos *Top Ten* de 2022. Ainda dá tempo! Submetam sua melhor ciência!
