# Supplementary material for: Os Melhores Artigos do Ano 2021 nos Arquivos Brasileiros de Cardiologia e na Revista Portuguesa de Cardiologia
Source: Arq Bras Cardiol. 2022 Jul 7;119(1):113–23. [Article in Portuguese] doi: 10.36660/abc.20220312 (PMC9352128; doi:10.36660/abc.20220312)
Supplement: Translation

# Translation: 2021 Top 10 Articles in the *Arquivos Brasileiros de Cardiologia* and the *Revista Portuguesa de Cardiologia*

**Authors:** Ricardo Fontes-Carvalho, Gláucia Maria Moraes de Oliveira, Pedro Gonçalves-Teixeira, Carlos Eduardo Rochitte, Nuno Cardim

**Affiliations:** 1 Vila Nova de Gaia Espinho Portugal Departamento de Cardiologia – Centro Hospitalar de Vila Nova de Gaia/Espinho, Vila Nova de Gaia/Espinho – Portugal; 2 Porto Portugal Cardiovascular Research Center (UniC), Faculty of Medicine, University of Porto, Porto, Portugal; 3 Rio de Janeiro RJ Brazil Faculdade de Medicina – Universidade Federal do Rio de Janeiro, Rio de Janeiro, RJ – Brazil; 4 Rio de Janeiro RJ Brazil Instituto do Coração Edson Saad – Universidade Federal do Rio de Janeiro, Rio de Janeiro, RJ – Brazil; 5 São Paulo SP Brazil Instituto do Coração (InCor) – Faculdade de Medicina da Universidade de São Paulo, São Paulo, SP – Brazil; 6 São Paulo SP Brazil Hospital do Coração (HCOR), São Paulo, SP – Brazil; 7 Lisboa Portugal Hospital da Luz, Lisboa – Portugal

**Keywords:** Brazil, Technical Cooperation/trends, Dissemination of Information, Cardiovascular Diseases, Impact Factor

## Introduction

Annually the *Revista Portuguesa de Cardiologia* (RPC) and the *Arquivos Brasileiros de Cardiologia* (ABC) have gathered to elaborate a special article with the selection of their best original publications.^1–3^ Over the years, the high quality of their papers has become clear, evidencing the dynamism of cardiovascular investigation in Portuguese-speaking countries.

Following tradition, the editorial bodies of the RPC and the ABC met once again to select the ten best articles published in 2021 by each of those journals (Tables 1 and 2). Although the year 2021 was marked again by the impact of the COVID-19 pandemic, both journals had excellent publications in all areas of Cardiovascular Medicine, from cardiovascular prevention to heart failure (HF), including an excellent selection of papers on the COVID-19 pandemic in the Brazilian and Portuguese populations. The high quality of the publications in both journals makes the selection of the best articles a complex task.

**Table 1:** List of the ten best articles published in the *Arquivos Brasileiros de Cardiologia* in 2021

Link	Authors and titles of the articles
https://abccardiol.org/wp-content/uploads/articles_xml/0066-782X-abc-117-01-0091/0066-782X-abc-117-01-0091.x44344.pdf	Alves P et al.
	Relação entre Resposta Imune Inata do Receptor Toll-Like-4 (TLR-4) e o Processo Fisiopatológico da Cardiomiopatia da Obesidade
	Relationship between Innate Immune Response Toll-Like Receptor 4 (TLR-4) and the Pathophysiological Process of Obesity Cardiomyopathy
https://abccardiol.org/wp-content/uploads/articles_xml/0066-782X-abc-116-06-1091/0066-782X-abc-116-06-1091.x44344.pdf	Morais TC et al.
	Performance Diagnóstica da FFR por Angiotomografia de Coronárias através de Software Baseado em Inteligência Artificial
	Diagnostic Performance of a Machine Learning-Based CT-Derived FFR in Detecting Flow-Limiting Stenosis
https://abccardiol.org/wp-content/uploads/articles_xml/0066-782X-abc-116-03-0466/0066-782X-abc-116-03-0466.x44344.pdf	Matos LCV et al.
	O Escore Gensini e a Carga Trombótica Adicionam Valor Preditivo ao Escore SYNTAX na Detecção de No-Reflow após Infarto do Miocárdio
	Gensini Score and Thrombus Burden Add Predictive Value to the SYNTAX Score in Detecting No-Reflow after Myocardial Infarction
https://abccardiol.org/wp-content/uploads/articles_xml/0066-782X-abc-117-05-0944/0066-782X-abc-117-05-0944.x44344.pdf	Santos SC et al.
	Mortalidade por Insuficiência Cardíaca e Desenvolvimento Socioeconômico no Brasil, 1980 a 2018
	Mortality Due to Heart Failure and Socioeconomic Development in Brazil between 1980 and 2018
https://abccardiol.org/wp-content/uploads/articles_xml/0066-782X-abc-117-03-0426/0066-782X-abc-117-03-0426.x44344.pdf	Santos IS et al.
	Diagnóstico de Fibrilação Atrial na Comunidade Utilizando Eletrocardiograma e Autorrelato: Análise Transversal do ELSA-Brasil
	Atrial Fibrillation Diagnosis using ECG Records and Self-Report in the Community: Cross-Sectional Analysis from ELSA-Brasil
https://abccardiol.org/wp-content/uploads/articles_xml/0066-782X-abc-116-02-0219/0066-782X-abc-116-02-0219.x44344.pdf	Mendes SL et al.
	Resultados Clínicos e Hemodinâmicos de Longo Prazo após o Transplante de Coração em Pacientes Pré-Tratados com Sildenafil
	Long-Term Clinical and Hemodynamic Outcomes after Heart Transplantation in Patients Pre-Treated with Sildenafil.
https://abccardiol.org/wp-content/uploads/articles_xml/1678-4170-abc-116-04-0695/1678-4170-abc-116-04-0695.x44344.pdf	Oliveira JC et al.
	Acesso à Terapia de Reperfusão e Mortalidade em Mulheres com Infarto Agudo do Miocárdio com Supradesnivelamento do Segmento ST: Registro VICTIM
	Access to Reperfusion Therapy and Mortality in Women with ST-Segment–Elevation Myocardial Infarction: VICTIM Register
https://abccardiol.org/wp-content/uploads/articles_xml/1678-4170-abc-116-04-0795/1678-4170-abc-116-04-0795.pdf	Hussid MF et al.
	Obesidade Visceral e Hipertensão Sistólica como Substratos da Disfunção Endotelial em Adolescentes Obesos
	Visceral Obesity and High Systolic Blood Pressure as the Substrate of Endothelial Dysfunction in Obese Adolescents
https://abccardiol.org/wp-content/uploads/articles_xml/0066-782X-abc-116-01-0004/0066-782X-abc-116-01-0004.x44344.pdf	Miguel-dos-Santos R et al.
	Treino de Força Reduz Stress Oxidativo Cardíaco e Renal em Ratos com Hipertensão Renovascular
	Strength Training Reduces Cardiac and Renal Oxidative Stress in Rats with Renovascular Hypertension
https://abccardiol.org/wp-content/uploads/articles_xml/0066-782X-abc-117-02-0309/0066-782X-abc-117-02-0309.x44344.pdf	Chehuen M et al.
	Respostas Fisiológicas à Caminhada Máxima e Submáxima em Pacientes com Doença Arterial Periférica Sintomática
	Physiological Responses to Maximal and Submaximal Walking in Patients with Symptomatic Peripheral Artery Disease
https://abccardiol.org/wp-content/uploads/articles_xml/0066-782X-abc-116-02-0266/0066-782X-abc-116-02-0266.x27815.pdf	Guimarães et al.
	Aumento de Óbitos Domiciliares devido a Parada Cardiorrespiratória em Tempos de Pandemia de COVID-19
	Increased Home Death Due to Cardiopulmonary Arrest in Times of COVID-19 Pandemic

**Table 2:** List of the ten best articles published in the *Revista Portuguesa de Cardiologia* in 2021

Authors	Titles of the articles
Dinis Mesquita et al.	Cardiac arrhythmias in patients presenting with COVID-19 treated in Portuguese hospitals: A national registry from the Portuguese Association of Arrhythmology, Pacing and Electrophysiology
Ana Manuel et al.	Long-term outcomes after radiofrequency catheter ablation of the atrioventricular node: The experience of a Portuguese tertiary center
Joana Ribeiro et al.	Impact of catheter ablation for atrial fibrillation in patients with heart failure and left ventricular systolic dysfunction
Jesús Velásquez-Rodríguez et al.	Influence of left ventricular systolic function on the long-term benefit of beta-blockers after ST-segment elevation myocardial infarction
Luis Raposo et al.	Adoption and patterns of use of invasive physiological assessment of coronary artery disease in a large cohort of 40 821 real-world procedures over a 12-year period
João Costa et al.	Atherosclerosis: The cost of illness in Portugal
Carina Silva et al.	Prognostic impact of iron deficiency in acute coronary syndromes
Luis Paiva et al.	Non-vitamin K antagonist oral anticoagulation versus left atrial appendage occlusion for primary and secondary stroke prevention after cardioembolic stroke
Catarina de Sousa et al.	The burden of infective endocarditis in Portugal in the last 30 years: a systematic review of observational studies
Felipa de Mello Sampayo et al.	Cost-effectiveness of cardio-oncology clinical assessment for prevention of chemotherapy-induced cardiotoxicity

We provide a list of the ten best articles in each journal and their brief description, as well as their major implications for cardiovascular disease diagnosis, treatment, and understanding. Aiming to improve their understanding, the articles were grouped and presented according to four general themes.

## COVID-19 and its consequences

The years 2020 and 2021 were marked by the huge impact of the COVID-19 pandemic on health care worldwide. In a study published in the RPC, Mesquita et al.^4^ have analyzed the prevalence and prognostic impact of cardiac arrhythmias on patients hospitalized with COVID-19. They used the national registry from the Portuguese Association of Arrhythmology, Pacing, and Electrophysiology with data from 20 Portuguese hospitals relative to 692 patients admitted due to COVID-19. Of those patients, 11.7% had arrhythmic events. The most frequent arrhythmias were atrial fibrillation and flutter (AFF – 62.5%). Two patients (3.1%) had ventricular tachycardia, and 17 (26.6%) had paroxysmal supraventricular tachycardia. Those patients had neither important complications from the arrhythmic event or death from arrhythmic cause, despite their more severe COVID-19 and many comorbidities, nor higher frequency of hemodynamic instability and/or multiple organ failure. Although 76.6% of the patients with arrhythmic events were on medication that increases the QT interval (ritonavir/lopinavir, hydroxychloroquine or azithromycin), only seven patients (10.9%) had a prolonged QT interval (ranging from 480ms to 596 ms).^4^ Those authors concluded that the incidence of cardiac arrhythmias is high in patients hospitalized due to COVID-19, but they did not associate with increased cardiac mortality, although they often occurred in patients with more severe disease and multiple organ failure. The incidence of ventricular arrhythmias was low although the patients were on medications that prolong the QT interval.

An observational study has assessed the mortality rate from cardiac arrest (CA) relative to the total number of household visits reported by the mobile urgent healthcare service (in Portuguese, SAMU) in the city of Belo Horizonte, Minas Gerais state, Brazil, in March 2018, 2019, and 2020. There was a gradual increase in the number of household deaths due to CA relative to the total number of visits provided by the SAMU, as well as a proportional 33% increase in household deaths in March 2020, when the COVID-19 pandemic began. Most patients (63.8%) were at least 60 years of age, and 87% of the reported CAs were associated with clinical comorbidities, such as systemic arterial hypertension (22.87%), HF (13.03%), and diabetes mellitus (11.0%). It is worth noting that, although the families knew the patients had comorbidities, in 38.4% of the cases reported, the families did not know which those comorbidities were.^5^ The Brazilian health system needs to better the patients’ and families’ knowledge about their diseases, emphasizing and improving access to the hospital system to reduce the impact of out-of-hospital CA, whose chance of survival is low.

Fernandes et al.^6^ have conducted a retrospective study with 187 patients admitted to an intensive care unit (ICU) after CA over a 5-year period. The median patients’ age was 67 years. In-hospital CA occurred in 61% of the cases, and 87% had an initial non-shockable rhythm. The mean time until return of spontaneous circulation (ROSC) was 10 minutes. Presumed cardiac causes accounted for only 31% of the cases, which is explained by the exclusion of patients with CA due to ST-elevation myocardial infarction (STEMI), who were directly admitted to the coronary ICU in the same hospital. Those authors reported in-hospital mortality of 63% (45% of which related to withdrawal of life-support measures) and 1-year mortality of 72%. The prevalence of favorable neurologic status at hospital discharge [cerebral performance category (CPC) = 1] was only 25%. The independent predictors of in-hospital mortality were time until initiating basic life support (BLS), high SAPS II score, non-shockable initial rhythm, and vasopressor support duration. Although the time until initiating BLS and the time until ROSC were longer for patients with out-of-hospital CA, the clinical results did not differ significantly in the two populations of patients. Survival with a favorable neurologic status (CPC = 1 or 2) was associated with less frequent epileptic activity and shorter ventilatory support, but not with assisted CA, initial rhythm, time until ROSC, or implementation of the normothermia protocol. Finally, the neurologic outcomes and mortality were similar in both sexes. That study emphasizes the importance of improving all chain of survival components to optimize those patients’ prognosis. In addition, it reinforces the need for clinical trials in the area, ideally multicenter and duly framed within the ethical context.^6^

### Cardiac arrhythmia and its impact on society

Atrial fibrillation and flutter are the most common arrhythmias in both the general population and the patients with COVID-19, although they do not associate with the presence of CA.^4–7^ Aiming to study the incidence of factors associated with cardiovascular diseases and diabetes, a longitudinal study of adult health in Brazil (ELSA-Brasil) was conducted. That study is a large multicenter cohort of individuals aged 35 to 74 years in six Brazilian cities. A substudy^8^ of the ELSA-Brasil, with 13 260 participants, investigated the presence of AFF, which was defined by use of electrocardiogram or self-report. That substudy aimed to assess whether age and sex were associated with the use of anticoagulants to prevent stroke. The authors reported predominance of the female sex (54.4%), median age of 51 years, and AFF detection in 333 (2.5%) participants. The authors reported the association of AFF with age (OR: 1.05; 95% CI: 1.04-1.07), arterial hypertension (OR: 1.44; 95% CI: 1.14-1.81), coronary artery disease (CAD - OR: 5.11; 95% CI: 3.85-6.79), HF (OR: 7.37; 95% CI: 5.00-10.87), and rheumatic fever (OR: 3.38; 95% CI: 2.28-5.02). Only 20 patients with CHA2DS2-VASc score ≥2 (10.8%) were on anticoagulants, whose use was smaller in women. Those findings represent a great challenge to the AFF treatment.

In 2018, the CASTLE-AF study^9^ showed that atrial fibrillation (AF) ablation could improve the prognosis of a selected population of patients with HF. In another study, Ribeiro et al.^10^ have retrospectively assessed the impact of AF ablation in 22 patients with HF (32% with NYHA functional class II and 58% with NYHA functional class III) and left ventricular ejection fraction (LVEF) <50%. The procedure was successful in 100% of the patients, with no complication reported. The recurrence of AF after the blanking period was 18%. After a median 11-month follow-up, those authors reported an improvement in functional capacity, with mean pre-procedure and post-procedure NYHA functional classes of 2.35±0.49 and 1.3±0.47, respectively, (p<0.001). In addition, the mean LVEF improved from 40% to 58% (p<0.01), and there was favorable remodeling of left cardiac chambers. The authors concluded that, in patients carefully selected with AF, HF and LVEF <50%, AF ablation results in significant functional class improvement, LVEF improvement, and favorable structural remodeling of left cardiac chambers. Similarly to large scale clinical trials recently published,^11^ those authors proposed AF ablation be early considered in those patients, because of its high safety profile as compared to pharmacological rhythm control strategies.

Regarding invasive electrophysiology, Manuel et al.^12^ have published an interesting study with long-term follow-up of patients submitted to radiofrequency catheter ablation of the atrioventricular node. They assessed data from 123 patients (mean age, 69±9 years) of a Portuguese tertiary center, with a median 8.5-year follow-up. The indications for that procedure were low percentage of biventricular pacing (8%), presence of tachycardiomyopathy (80%, and 65% of which related to AF of rapid ventricular response despite pharmacological therapy), the occurrence of inappropriate shocks of implantable cardiac defibrillator (2%) or uncontrollable supraventricular tachycardia (10%). In 89% of the cases, a device was implanted during ablation, 14% of which were of cardiac resynchronization, while the remaining patients already carried such devices. The procedure was successfully performed in all patients, with no periprocedural major complication. It is worth noting that, during follow-up, no major complication was documented in association with implantable cardiac devices. In addition, the authors reported improvement of the NYHA functional class, fewer hospitalizations, and a reduction in the number of unplanned visits due to HF decompensation. All-cause mortality was 3.5% by the end of the first year and 23% throughout the entire follow-up, in accordance with that reported in the literature. That study shows the high efficacy and safety of the atrioventricular node ablation procedure in selected patients, a technique that can be especially important to treat complex patients, allowing symptom improvement and a reduction in hospitalization from HF, as reported in the European guidelines.^13^

Left atrial appendage occlusion (LAAO) is a current controversial theme in cardiology. In a study published in the RPC, Paiva et al.^14^ have analyzed the safety and efficacy of that procedure in patients with nonvalvular AF for primary and secondary stroke prevention. Those authors have conducted a prospective observational study involving 91 patients submitted to LAAO and 149 patients treated with nonvitamin K antagonist oral anticoagulants (DOACs) in a mean 13-month follow-up. The mean age of the patients undergoing LAAO was 74.7±8.7 years (vs. 77.8±8.0 years of the patients on DOACs), 59% were of the male sex. Their mean CHA2DS2-VASc score was 4.3±1.4 (vs. 5.3±1.3) and their mean HAS-BLED score was 3.0±0.9 (vs. 4.0±0.7). The devices ACP/Amulet™ and Watchman™ were used, and the procedural rate of success was 96.3%, with no major complication reported. The study showed a nonsignificant trend towards a reduction in the composite endpoint of death, stroke, and major bleeding in patients submitted to LAAO (11.0% vs. 20.9%; HR: 0.42, 95% CI: 0.17-1.05, p=0.06). Approximately 20% of the patients submitted to LAAO stopped antiplatelet treatment six months after device implantation due to recurrent minor bleeding, but neither cardiovascular events nor severe bleedings occurred. The authors concluded that LAAO was not inferior to DOACs to prevent the primary composite endpoint of death, stroke, and major bleeding in patients with nonvalvular AF. The scientific community awaits the results of several ongoing randomized clinical trials on this subject, which might provide definitive responses regarding the role of LAAO in the treatment of patients at high risk for stroke (for example, patients with history of intracranial bleeding).^15–18^

The major question regarding LAAO is its cost-effectiveness, yet to be answered in the literature available. In fact, health economics has become one of the most relevant themes, especially in countries that provide universal access to the health system, such as Brazil. A good example is the cost associated with atherosclerosis, which is the pathological denominator common to the major causes of morbidity and mortality in developed countries, such as acute and chronic coronary syndromes, ischemic stroke, and peripheral artery disease. In an article published in the RPC, Costa et al.^19^ tried to quantify the economic impact of atherosclerosis in Portugal, using prevalence data and recurring to multiple national databases. The total costs of atherosclerosis in 2016 reached 1.9 billion euros, which correspond to 11% of all health expenditure and to approximately 1% of the gross domestic product in 2016. Of those costs, 58% represented direct costs with the disease, 55% of which related to primary health care, and 42% were indirect costs, 91% of which related to labor absenteeism. Of the manifestations of atherosclerosis, ischemic heart disease had a higher cost per patient, mainly due to medication cost. One may conclude that, considering its high prevalence (9% of the adult population in Portugal) and economic impact, atherosclerosis remains a clinical, social, and financial challenge to the health systems around the world, which may be compounded in the future by population aging.

### Current challenges in coronary artery disease

The higher cost of atherosclerotic disease per patient relates to ischemic heart disease and its approach, mainly because of the different therapies available.^19^ Several studies have shown that invasive coronary functional assessment with fractional flow reserve (FFR or iFFR) can be cost-effective^20^ and improve the prognosis.^21–23^ However, its use in clinical practice is still residual in most interventional cardiology laboratories. In an interesting study, Raposo et al.^24^ have assessed the pattern of use of those techniques of invasive functional assessment of CAD in two reference centers over a period of 12 years, with a total of 40 821 patients submitted to invasive coronary angiography. Those techniques were applied to only 0.6% of the patients undergoing coronary angiography for valve disease and to 6.0% of those undergoing coronary angiography for stable CAD. In 42.9% of the stable CAD patients undergoing percutaneous coronary intervention (PCI), neither there was evidence of previous ischemia on imaging tests nor physiological assessment was performed. The age of the operators and the time of procedure associated significantly with the use of invasive physiology. The publication timing of reference clinical trials and of relevant international recommendations associated with a higher rate of adoption of those techniques. The scientific evidence on non-hyperemic indices (iFFR), of easy and rapid use, increased the proportion of their use as compared to hyperemic indices (FFR), however without increasing the overall rate of use. The authors concluded that the suboptimal rate of use of the invasive functional assessment of CAD is an opportunity to improve the prognosis of patients with angiographically moderate CAD through dedicated strategies aimed at increasing adherence to scientific recommendations and reducing clinical inertia.

Noninvasive quantification of myocardial FFR can be particularly useful in moderate stenoses (50% to 69%), helping discriminate lesions associated with significant ischemia.^25^ Recent studies have shown the high accuracy of noninvasive FFR computed from coronary computed tomography angiography (FFR_CT_) to identify myocardial ischemia as compared to FFR or iFFR, considered the gold-standard method.^26,27^ A retrospective study with patients referred for coronary computed tomography angiography and coronary angiography has assessed the diagnostic performance of FFR_CT_ in detecting significant CAD as compared to FFR, defining obstructive CAD as lumen reduction ≥50% on coronary computed tomography angiography and functionally obstructive CAD as FFR ≤0.8. The study included 93 consecutive patients (152 vessels) and assessed FFR_CT_ by using a machine-learning-based software. Good agreement regarding the FFR measure was observed, with a post-processing time of 10 minutes. Regarding diagnostic performance, even in previous generation CT scanners, there was good agreement between FFR_CT_ and FFR, with minimal FFR_CT_ overestimation (bias: -0.02; limits of agreement: 0.14-0.09). The FFR_CT_ *performance was* significantly superior as compared to the visual classification of coronary stenosis, reducing the number of false-positive cases. The authors have concluded that artificial-intelligence-based FFR_CT_, even using previous generation CT scanners, shows good diagnostic *performance for* CAD detection, which can be used to reduce invasive procedures.^28^

According to data from the Brazilian Unified Public Health System (SUS), the number of hospitalizations from MI in the public health system adjusted to population increased by 54% from 2008 to 2019. The number of nonprimary PCI procedures per inhabitant doubled, while that of primary PCI increased by 31%. The in-hospital mortality from MI decreased from 15.9% in 2008 to 12.9% in 2019, which is still very high as compared to the world rates.^7^ It is worth noting that access to myocardial reperfusion, which is the therapeutic basis of MI, is not widely available, especially for women, increasing mortality and impacting on the total costs of atherothrombotic disease. A cross-sectional study with data from the VICTIM Registry has assessed patients diagnosed with STEMI from four hospitals (one public and three private) that offer primary PCI in the state of Sergipe, from December 2014 to June 2018. The study included 878 patients with STEMI, 33.4% of whom were women. Slightly more than half of the patients underwent myocardial reperfusion (53.3%, 134 women and 334 men). Women had lower rates of fibrinolysis (2.3% of the total, 1.7% in women and 2.6% in men) and of primary PCI (44% in women and 54.5% in men), resulting in higher in-hospital mortality for women as compared to that for men (16.1% vs. 6.7%). It is worth noting that women, as compared to men, had higher rates of diabetes mellitus (42% vs. 28.5%), systemic arterial hypertension (75.1% vs. 59%), and dyslipidemia (50.2% vs. 33.3%). The authors have emphasized the worst results for the public health system users, mainly for women, and pointed to the need to improve the access of women with STEMI to effective treatment strategies to reduce in-hospital mortality.^29^

Primary PCI is aimed at restoring arterial lumen patency to promote blood flow in coronary microcirculation. However, one in every three patients remains with reduced microvascular flow even with restored macrovascular flow, a phenomenon known as no-reflow. Those patients are at higher risk for HF, cardiogenic shock, and cardiovascular death.^30^ Although the SYNTAX score is a good predictor of microvascular dysfunction, the atherosclerotic and thrombus burdens are not considered in the algorithm, because of the exclusion of obstructive lesions with stenoses <50% and the attribution of a low score to the presence or absence of a thrombus, respectively. To assess whether the atherosclerotic burden (through the Gensini score) and the thrombus burden in the culprit coronary artery improved the ability of the SYNTAX score to detect no-reflow, Matos et al.^31^ have assessed 481 consecutive patients with STEMI, who presented within 12 hours from symptom onset. No-reflow was defined as TIMI flow <3 or TIMI flow =3, but myocardial blush grade <2. Thrombus burden was quantified according to the TIMI thrombus grade scale (0 to 5). Patients’ mean age was 61±11 years, and no-reflow occurred in 32.8% of the patients. The independent predictors of no-reflow were SYNTAX score (OR=1.05, 95% CI: 1.01–1.08, p<0.01), thrombus burden (OR=1.17, 95% CI: 1.06–1.31, p<0.01), and Gensini score (OR=1.37, 95% CI: 1.13–1.65, p<0.01). The combined scores had a larger area under the curve as compared to the SYNTAX score alone (0.78 [0.73–0.82] vs. 0.73 [0.68–0.78], p=0.03). Those authors have concluded that the atherosclerotic and thrombus burdens in the culprit artery add predictive value to the SYNTAX score to detect no-reflow. The major limitation of that study is its cross-sectional and single-center character.

One strategy to improve mortality from MI would be the use of beta-blockers (BB) and acetylsalicylic acid. Recently the long-term prognostic benefit of BB use after acute coronary syndrome has been questioned. In a retrospective study published in the RPC, Velásquez-Rodríguez et al.^32^ have tried to respond that question analyzing 972 consecutive patients admitted with STEMI, 99.7% of whom submitted to PCI, with a mean age of 62.6±13.5 years, and 21.8% of the female sex. At discharge, 85.9% of the patients were on BB. As expected, those who did not receive BB had more comorbidities (neoplasia, anemia, chronic obstructive pulmonary disease), and higher prevalence of inferior STEMI and of high-grade atrioventricular block. After a mean follow-up of 49.6±24.9 months, the use of BB was an independent predictor of survival in the general population (HR 0.61, 95% CI: 0.38-0.96), but, when stratified according to LVEF, only those with LVEF ≤40% seemed to have a survival benefit with that therapy. The authors have concluded that the study results raise reasonable doubts regarding the real benefit of the long-term systematic use of BB after STEMI in patients with LVEF >40%. In another observational study including 1520 patients, published in the RPC in 2018, Timóteo AT et al.^33^ concluded that the systematic use of BB after acute coronary syndrome was beneficial regardless of the LVEF value. Briefly, in 2022 it seems reasonable to state that, in the absence of scientific evidence from randomized clinical trials with proper sample sizes, there is still room for BB therapeutic individualization after MI in patients with LVEF >40% that considers the patients’ mean life expectancy, their preferences, functional and cognitive status, comorbidities, frailties, drug interactions, and adverse reactions.^34^

New strategies to minimize adverse outcomes from MI are welcome, one of them being the extrapolation of data from patients with HF of ischemic etiology.^35^ In the literature, data relative to the prognostic impact of iron deficiency in acute coronary syndromes are scarce. A study published in the RPC by Silva et al.^36^ has aimed to fill that gap. That study assessed data from 817 patients admitted due to acute coronary syndrome to a Portuguese tertiary hospital. The patients were assigned to two study groups according to the presence (n=298, 36%) or absence (n=519) of iron deficiency on admission. Those with iron deficiency more frequently had moderate and severe left ventricular dysfunction, right ventricular dysfunction, and higher Killip classes. In the middle-term follow-up (mean of 738 days), those patients showed higher all-cause mortality (12.6% vs. 6.3%, p=0.04), NYHA functional class III/IV HF (10.5% vs. 5.3%, p=0.011), as well as higher rate of hospital readmission (9.8% vs. 13.7%, p=0.048). Iron deficiency was an independent predictor of death or HF during follow-up and enabled the prognostic stratification, regarding the occurrence of death or HF, of patients without anemia and/or with lower Killip classes (≤2). The authors concluded that iron deficiency is not only a prevalent condition among patients with acute coronary syndrome but also an independent predictor of death or severe HF in the middle-term follow-up. In addition, iron deficiency can be an interesting complement in the prognostic stratification of patients with acute coronary syndrome without anemia, as well as in those with Killip classes ≤2.

### Heart failure: from causes to prevention

Heart failure affects approximately 26 million people around the world and these figures tend to increase with population aging, high prevalence of cardiovascular risk factors, patients’ survival from MI, and improvement in HF therapies. In addition, worse outcomes from HF and social determinants seem to be associated.^37^ Santos et al.^38^ have assessed the temporal progression of the mortality rates from HF according to sex and age group in Brazil, its geographic regions, and federative units, from 1980 to 2018, and their association with the Municipal Human Development Index (MHDI). Mortality from HF decreased in Brazil over the 29 years studied, with a trend towards progressive reduction since 2008, reaching, by the end of 2018, similar levels in the geographic regions and federative units. The mortality rates from HF in the male sex were higher almost in all periods and age ranges assessed, probably because of the relation with the ischemic etiology of HF. An inverse trend was observed between the variation in the mortality rates of the federative units from 1990 to 2018 and the variation in their respective MHDI from 1991 to 2010. The authors suggested that, regarding mortality from HF, more important than the increase in the MHDI is the final level of that index (MHDI ≥0.7). The authors concluded that the access to health care should be expanded and encouraged a more effective control of the cardiovascular risk factors (dyslipidemia, obesity, sedentary lifestyle, diabetes) and of the social determinants, which contribute significantly to mortality from ischemic heart disease and HF.

Considering the increasing population at risk for developing HF after exposure to cardiotoxic chemotherapy (CTX), in addition to the scarce scientific evidence to support different strategies of cardioprotection in those patients, Mello et al.^39^ have published a cost-effectiveness study to provide some answers. Those authors have calculated and compared the QALYs of two cardioprotective strategies: a universal cardioprotective strategy (all patients receiving BB and angiotensin-converting-enzyme inhibitor); and another guided by LVEF imaging assessment (in which the cardioprotective medication was initiated upon diagnosis of HF due to CTX, defined by a symptomatic LVEF decrease >10% to a final value ≤55%). For that calculation, a Monte Carlo simulation of a Markov model was used. The authors concluded that, from a cost-effectiveness perspective, the imaging-assessment-guided cardioprotective strategy was superior to the universal cardioprotective strategy, providing more QALYs at a lower cost. In the reference case of a 63-year-old female with breast cancer undergoing CTX with anthracyclines and trastuzumab over 5 years, the imaging-assessment-guided cardioprotective strategy resulted in 4.22 QALYs at a cost of €2594, while the universal cardioprotective strategy resulted in 3.42 QALYs at a cost of €3758. Further large-scale clinical trials are needed to better define the population of patients who benefit from cardioprotective strategies in cardiotoxicity primary prevention.

The obesity-related disorders, such as insulin resistance, diabetes, and dyslipidemia, are considered HF predictors and associate with adipose tissue dysfunction, promoting maladaptive cardiac responses, such as myocyte hypertrophy, contractile dysfunction, and cardiac remodeling, which contribute to their development and chronic HF progression.^40^ Alves et al.,^41^ in an elegant study with Wistar rats, have hypothesized that the activation of the toll-like receptor 4 (TLR-4) participates in the obesity-related cardiac disease by triggering cytokine production via nuclear factor-ĸB (NF-ĸB). The ‘obese’ group, which was fed a high sugar-fat diet and water plus 25% of sucrose for 30 weeks, showed: obesity, high levels of glucose, triglycerides and uric acid, insulin resistance, high systolic blood pressure, high levels of tumor necrosis factor alpha (TNF-α) in the adipose tissue, in addition to cardiac remodeling and diastolic dysfunction. In the obese group of Wistar rats, the TLR-4 and NF-ĸB expression, the levels of cytokines, and the TLR-4 gene and protein expression were higher, and the NF-ĸ B phosphorylation was increased, confirming the activation of that pathway as an inflammation mediator. The authors concluded that the innate immune response via TLR-4 activation is one of the mechanisms that can contribute to the myocardial inflammatory process of obesity, and that the inflammation derived from cardiac TLR-4 activation is a new mechanism that can lead to cardiac remodeling and dysfunction.

Obesity in adolescence can lead to metabolic syndrome (MS) and endothelial dysfunction, being a predictor of adult obesity, in addition to an early marker of cardiovascular risk. It is worth noting that sleep respiratory diseases, such as obstructive sleep apnea (OSA), are some of the consequences of obesity.^42^ To investigate whether obesity in adolescence is associated with MS and/or OSA, in addition to its relation to endothelial dysfunction, Hussid et al.^43^ have studied 20 sedentary obese adolescents (14.2±1.6 years, 100.9±20.3 kg) and 10 normal-weight adolescents (15.2±1.2 years, 54.4±5.3 kg) paired by sex. The authors assessed the risk factors for MS, vascular function (flow-mediated dilation), functional capacity (VO_2_peak), and the presence of OSA (apnea-hypopnea index > 1 event/hour on polysomnography). In the sample studied, obesity was an important risk factor for the MS development and led to endothelial dysfunction. Increased waist circumference and systolic blood pressure were predictors of endothelial dysfunction in adolescents. Most adolescents had OSA, regardless of obesity. The reduced sample size, girls’ menstrual cycle hormones affecting the endothelial function, and absence of patterns for the variables studied in the Brazilian adolescents might have influenced the study’s findings.^43^

Heart transplantation (HT) is the gold-standard care of terminal HF and there is a strong association between pulmonary artery systolic pressure (PASP) and lethality from that procedure, severe pulmonary hypertension (PH) being one of the major contraindications for transplantation because of post-operative right heart dysfunction.^44^ Mendes et at.^45^ have studied 300 consecutive candidates for HT treated between 2003 and 2013, dividing them into two groups according to the presence of PH. Of those patients, 95 had fixed PH, 30 of whom were pre-treated with sildenafil and received a HT, constituting group A. Group B included 205 patients without PH who also received a HT. The PASP decreased after HT in both groups but remained significantly high in group A as compared to that in group B (40.3 ± 8.0 mm Hg vs. 36.5 ± 11.5 mm Hg, p=0.022). One year after HT, PASP was 32.4 ± 6.3 mm Hg in group A *versus* 30.5 ± 8.2 mm Hg in group B (p=0.274). The authors concluded that, in patients with PH pre-treated with sildenafil, early post-operative hemodynamics and prognosis are numerically worse as compared to those in patients without PH. However, after 1 year, middle- and long-term mortalities are similar. They emphasized that the use of sildenafil to reduce PASP can be considered a valuable ‘rescue therapy’ in the group of patients with terminal HF.

Physical exercises minimize the major HF determinants, such as obesity, hypertension, and myocardial ischemia, and help both primary and secondary prevention of atherothrombotic disease. Patients with symptomatic peripheral artery disease (PAD), especially with intermittent claudication, have arterial hypertension, autonomic cardiac dysfunction, endothelial dysfunction, and increased oxidative stress and inflammation. In addition, PAD is a marker of atherosclerotic burden and associates with multisite atherothrombotic disease. Exercise training is considered the best treatment for patients with symptomatic PAD because it improves those patients’ locomotion ability, claudication symptoms, quality of life, and cardiovascular health.^46^ Cheuen et al.^47^ have conducted a study to compare the acute effects of maximal and submaximal walking on cardiovascular function and to assess the post-exercise regulation and associated pathophysiological processes in patients with symptomatic PAD. The authors recruited 30 men who underwent two sessions: maximal walking (Gardner’s protocol) and submaximal walking (15 bouts of 2 minutes of walking separated by 2 minutes of upright rest). In each session, the following parameters were assessed before and after walking: vital signs, heart rate variability, forearm and calf blood flows, reactive hyperemia, lipid peroxidation, and plasma levels of nitric oxide, C-reactive protein, TNF-α, and vascular and intercellular adhesion molecules (VCAM and ICAM, respectively). They reported that submaximal, but not maximal, walking reduced post-exercise blood pressure, while maximal walking maintained cardiac overload elevated during the recovery period. The maximal and submaximal walking sessions increased heart rate, cardiac sympathovagal balance, and post-exercise inflammation similarly, but did not change post-exercise nitric oxide bioavailability and oxidative stress. New longitudinal studies with larger and diverse populations need to be conducted to measure the long-term effects and to establish the best approach to patients with symptomatic PAD.

Still regarding physical exercise, Santos et al.^48^ have studied male Wistar rats, aged 10 to 12 weeks, to assess whether strength training reduces oxidative damage to the heart and contralateral kidney caused by renovascular hypertension induction surgery. In addition, they assessed the changes in the activity of endogenous antioxidant enzymes (superoxide dismutase, catalase, and glutathione peroxidase). The strength training was initiated four weeks after renovascular hypertension induction, was performed at 70% of 1RM, and lasted for 12 weeks. After the training period, there was a reduction in oxidative damage to lipids and proteins, with reduction in the hydroperoxides and total sulfhydryl levels, respectively, as well as an increase in the activities of superoxide dismutase, catalase, and glutathione peroxidase. Those authors concluded that the strength training can reduce oxidative damage by increasing the activities of antioxidant enzymes and suggested that strength training can be a nonpharmacological tool to treat renovascular hypertension, with the potential to prevent the damage advance to the heart and the kidney without renal artery stenosis. Studies on human beings need to be performed to confirm the hypothesis.

Sousa et al.^49^ have published a study on the epidemiology of infective endocarditis in Portugal, a systematic review of observational studies, including data from 1872 patients and enabling the assessment of important trends in diagnosis, treatment, and prognosis of those patients over the past three decades. The mean age was 55.5 ± 12.1 years, and there was a significant prevalence of males. Over time, the series reported a trend towards an increase in the patients’ mean age, which was 61.6 ± 16.3 years in 2008. The percentage of patients with endocarditis of a prosthetic valve or of an implantable cardiac device has also increased over the past decades, being 22.6% and 6.0%, respectively, in the most recent series. The prevalence of enterococcal infection was 10.2%, in accordance with data from the EURO-ENDO European registry^50^ and once again reflecting the patients’ growing aging. The use of non-ultrasonographic cardiac imaging techniques (FDG-PET and cardiac computed tomography angiography) was globally low because most patients were treated before the 2015 European guidelines,^51^ in which assessment with those techniques played a major role for the first time in the diagnosis and prognostic stratification. The rate of cardiac surgery varied substantially (3.1% to 52%), being higher in the most recent series. Short-term mortality ranged from 3.0% to 37.2%. That study provides important epidemiological information on a pathology of growing prevalence and complexity. This information can encourage clinical trials about the subject, on which scientific evidence regarding the best diagnostic and therapeutic strategies is still scarce.

## Conclusions

Despite the COVID-19 pandemic, 2021 was a year of intense activity for scientific publication in the *Arquivos Brasileiros de Cardiologia* and the *Revista Portuguesa de Cardiologia*. In this review of the ten best articles of 2021 in each of those journals, we aimed to review in a clear and practical way the findings of those papers and to emphasize their clinical importance for the understanding of cardiovascular disease. It is paramount to acknowledge how we advanced last year so we can progress even more in the coming years.

The 2021 highlights were for the thematic areas as follows: COVID-19 and its consequences, cardiac arrhythmia and its impact on society, current challenges in coronary artery disease, and heart failure: from causes to prevention. The articles approached new technologies, epidemiology of diseases, experimental studies for pathophysiological understanding, and fight of risk factors through a healthy lifestyle, particularly with studies on physical exercise and its role in cardiovascular diseases.

It was a true honor and pleasure for the editors of the two most important scientific journals in Portuguese to comment on such high-quality articles. We are very grateful to all authors who continue to submit their best science to our journals. We hope our readers enjoy this review and be encouraged to have their own articles in the list of the 2022 Top Ten. It is not too late! Submit your best science!
